# Air Embolism after Endoscopic Retrograde Cholangiopancreatography in a Patient with Budd Chiari Syndrome

**DOI:** 10.1155/2014/205081

**Published:** 2014-11-18

**Authors:** Beatriz Wills-Sanin, Yenny R. Cárdenas, Lucas Polanco, Oscar Rivero, Sebastian Suarez, Andrés F. Buitrago

**Affiliations:** ^1^Department of Critical Care, University Hospital Fundación Santa Fe de Bogotá, Calle 119 No. 7-75, A.A. 220246, Bogotá, Colombia; ^2^University Hospital Fundación Santa Fe de Bogotá, Calle 119 No. 7-75, A.A. 220246, Bogotá, Colombia

## Abstract

Endoscopic retrograde cholangiopancreatography is a procedure commonly used for the diagnosis and treatment of various pancreatic and biliary diseases. Air embolism is a rare complication, which may be associated with this procedure. This condition can be manifested as cardiopulmonary instability and/or neurological symptoms. Known risk factors include: sphincterotomy; application of air with high intramural pressure; anatomic abnormalities; and chronic hepatobiliary inflammation. It is important for the health-care staff, including anesthesiologists, interventional gastroenterologists, and critical care specialists, amongst others, to promptly recognize air embolism and to initiate therapy in a timely fashion, thus preventing potentially fatal outcomes. We submit a brief review of the literature and a case report of air embolism which occurred in the immediate postoperative stage of an endoscopic retrograde cholangiopancreatography, performed in a woman with a history of liver transplantation due to Budd Chiari syndrome and biliary stricture.

## 1. Introduction

Endoscopic retrograde cholangiopancreatography (ERCP) has become a primary tool for the diagnosis and treatment of ductal pancreatic and biliary tree pathology. Due to its complexity and technical demands, the endoscopic technique has a higher rate of complications than other diagnostic procedures [1], some of which include post-ERCP pancreatitis, cholangitis, bleeding or perforation after sphincterotomy. However, endoscopists are familiar with these adverse events, making ERCP a minimally invasive procedure with a good safety profile. Nonetheless, other less common situations—such as air embolism—can result in fatal outcomes. Therefore, it is essential for health professionals to identify risk factors and clinical manifestations of post-ERCP air embolism to improve its management [2].

## 2. Case Presentation

A 55-year-old female with a history of liver transplant six years ago due to Budd Chiari syndrome is admitted to the emergency room with progressive right-upper-quadrant pain, vomit and diarrhea. Her history included multiple episodes of biliary strictures managed with a self-expanding coated stent implant. At admission, her vital signs were stable, she had adequate oxygen saturation, and she had no fever. Lab reports evidenced elevated alkaline phosphatase and normal bilirubin levels.

**Figure 1:**
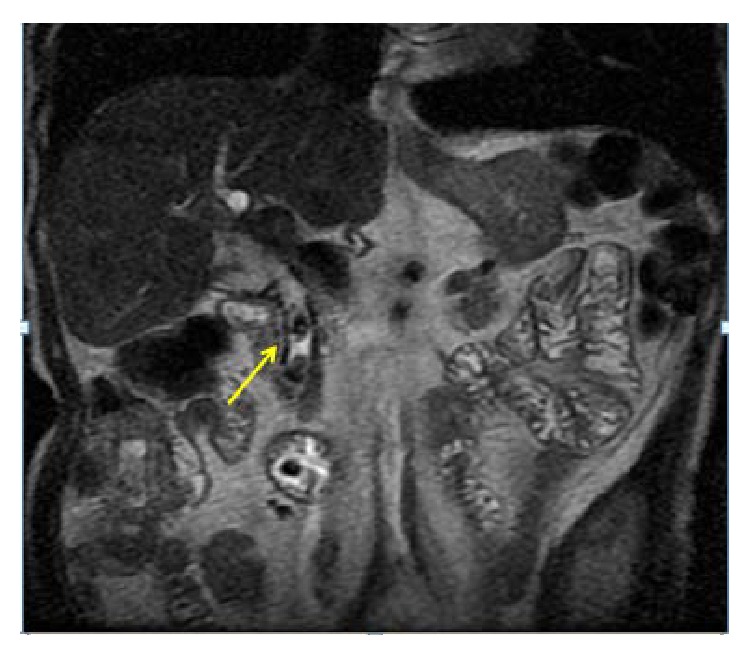
*Magnetic resonance* cholangiopancreaticography showing obstructive biliary processes associated with stent dysfunction of the extrahepatic and common bile duct. Intraluminal irregular images (yellow arrow) of low signal intensity on T2-weighted sequences consistent with bile stones are observed; these lead to moderate dilation of the intrahepatic bile duct.

To evaluate a probable biliary obstructive syndrome, an abdominal ultrasound and a magnetic resonance cholangiopancreatography were performed (Figure 1), which showed obstruction of the bile duct, secondary expansion, and presence of multiple gallstones. Due to the high risk of biliary sepsis and probable stent dysfunction, the patient was hospitalized and assessed by interventional gastroenterology, who decided to perform an endoscopic retrograde cholangiography for exchange of biliary stent and stone extraction.

During the procedure, prior papillotomy was identified and biliary stent migration and blockage by small stones were observed. These were removed under guidance with a balloon extractor. Uncoupling between bile duct receptor (8 mm) and donor (20 mm) persisted. Abdominal X-ray confirmed almost complete drainage of contrast media.

**Figure 2:**
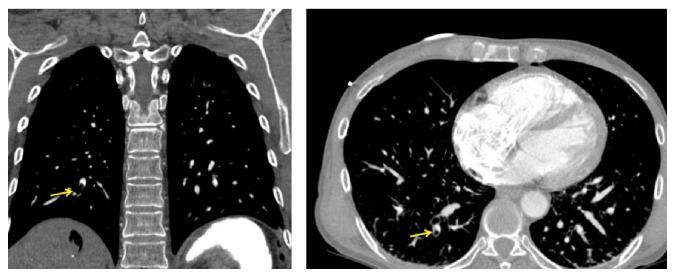
Contrast enhanced coronal and axial chest CT. Subsegmental artery air embolism in apical segment of right lower lobe surrounded by contrast media is observed (yellow arrows).

**Figure 3:**
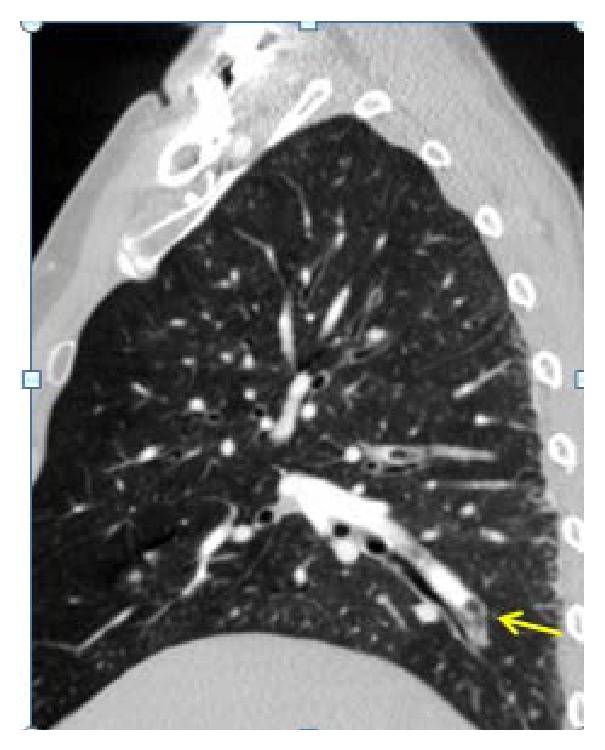
Contrast enhanced sagittal chest CT. Intravascular filling defect with air density bubble in subsegmental artery (yellow arrow) of apical segment of right lower lobe, bronchus is underneath.

**Figure 4:**
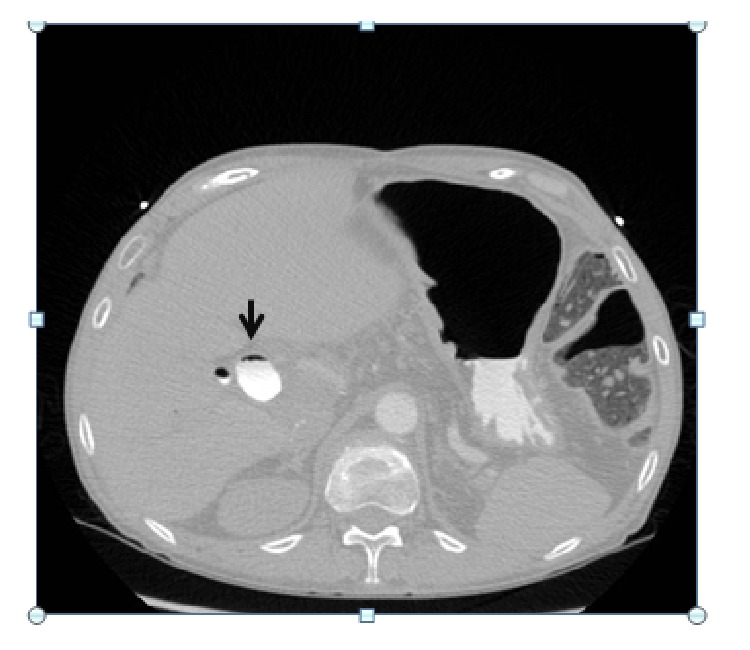
Contrast enhanced axial CT view showing air embolism (black arrow) inside intrahepatic portion of vena cava.

In the immediate postoperative period, the patient had hemodynamic collapse with pulseless electrical activity. Life support maneuvers followed and, after 2 minutes, spontaneous circulation was restored. Emergency transthoracic echocardiography revealed IVC collapse, with preserved ejection fraction without signs of cardiac tamponade. A CT angiography was done to rule out massive thromboembolism, which evidenced air embolus in the subsegmental arteries of the apical segment of the right lower lobe (Figures 2 and 3) and gas within the IVC in its hepatic portion (Figure 4).

The patient was admitted to the intensive care unit (ICU) with blood pressure of 76/46 mmHg, mean arterial pressure of 51 mmHg, heart rate of 64 bpm, oxygen saturation of 84%, rhythmic heart sounds, no murmurs, and paleness, requiring high concentrations of vasopressors. Symmetric expansion of chest under controlled assisted ventilation mode was indicated with the following parameters: respiratory frequency 18, 50% FiO_2_, PEEP 6, peak pressure 12 cm H_2_O, mean pressure 8.4, relationship inspiration: expiration 1 : 2.3, and tidal volume 352 mL. The patient continued receiving medical treatment and, after a favorable clinical outcome, she was discharged of the ICU 48 hours later.

### 2.1. Definition and Diagnosis

Air embolism subsequent to an invasive procedure is a rare adverse event which can lead to serious long-term neurological deficits or cause fatal cardiopulmonary compromise [3].

The symptoms of air embolism associated with ERCP occur or worsen when the patient is repositioned from prone to supine. The diagnosis is confirmed after visualizing air in the vena cava, portal vein, hepatic veins, right atrium, right ventricle, left atrium, left ventricle, or brain using different radiological aids such as transthoracic or transesophageal echocardiography or chest X-ray. However, the diagnosis of an air embolism can be complex, not only because of its rarity and lack of clinical suspicion, but also because the air can be absorbed rapidly from the circulation.

Air embolism may be confused with anesthetic side effects or with an acute ischemic or hemorrhagic event. Clinical manifestations of air embolism can be cardiovascular, including arrhythmia, hypotension, myocardial ischemia, right heart failure, cardiovascular collapse, and/or cardiac arrest. While sudden dyspnea, tachypnea, rales, wheezing, decreased final concentration of expired carbon dioxide, hypoxia, or cyanosis may confirm respiratory compromise. Neurological impairment such as eye deviation, mydriasis, altered state of consciousness during anesthetic recovery, hypertonia, cerebral hypoperfusion, cerebral edema and coma can also be present [4].

### 2.2. Pathophysiology

Air embolism is caused by a direct communication between a source of air and the circulation; it is favored by the pressure gradient that facilitates the passage of air into the circulation. The effect of an air bubble depends on both the flow and the volume of air introduced into the circulation. Venous air embolism occurs when air enters the systemic venous circulation, whereas arterial air embolism can lead to tissue ischemia [5].

Different mechanisms explaining air inlet to the venous system post ERCP have been proposed: (i) intramural dissection by the air blown into the portal vein; (ii) transection of duodenal veins; (iii) fistulas; (iv) portocaval collateral circulation; (v) air directed into the hepatic veins or into the inferior vena cava (IVC); (vi) retrograde flow through the superior vena cava (SVC); (vii) inability of the pulmonary circulation to filter gaseous emboli [6].

Irritation of the bile duct wall also has an important etiologic role in post-ERCP air embolism. Injury may be secondary to endoscopic instrumentation, gallstones, contrast media, stent material, or chronic inflammation. All these factors promote the development of a biliary-venous shunt, serving as a gateway for air.

Other authors have proposed that air embolism may be the result of pressure damage to the biliary tree mucosa caused by the air blown into the cavities. Similarly, preexisting biliovenous shunts can be interrupted by the increased pressure or due to bacteremia. Another mechanism that can explain air embolism after ERCP is the transfer of air through the bile duct wall or sphincter to the adjacent veins. Broken or expanded sutures from a previous surgery could also allow air passage [7].

Rapid entry or large volumes of air entering the systemic venous circulation cause a major strain on the right ventricle. This causes a significant increase in pulmonary arterial pressure, leading to obstruction of right ventricular outflow and decreased pulmonary venous return. This results in a decreased left ventricular preload with a consequent decrease in cardiac output, which eventually leads to cardiovascular failure. This was the case of our patient, where collapse of the vena cava led to cardiovascular compromise, which fortunately responded to resuscitation.

Venous air embolism may be limited to the portal venous system, or it may progress into a systemic air embolism via intracardiac or intrapulmonary shunting. It can also be caused by retrograde flow to the cerebral veins through the SVC, or by passage into the left atrium through the pulmonary veins. Since the most common cause of intracardiac shunt is a patent foramen ovale, it is critical to obtain a detailed clinical history in order to anticipate and identify potential complications.

In the case we report herein, several mechanisms may have contributed to the air embolism. First, Budd Chiari and multiple biliary stenosis episodes cause chronic inflammation that may alter gallbladder wall stability and favor the creation of venous dissection and the need of instrumentation procedures such as sphincterotomy. Other risk factors that may have contributed include a possible gateway through a peripheral or central venous line; prolonged exposure to high pressure blowing air; and mechanical damage during the biliary stent deployment of biliary dilatation [8].

### 2.3. Risk Factors

Most cases of air embolism related to endoscopy have been associated with ERCP and, of our knowledge, only 27 cases of air embolism after ERCP have been reported. Risk factors included cholangioscopy with air insufflation directed into the bile duct or with high pressure, hepatobiliary surgery, portosystemic shunts, percutaneous biliary drainage, penetrating liver trauma, history of large biliary, metal stent, inflammation of the bile duct or adjacent veins, liver abscesses or tumors, recent liver biopsy, altered papillary anatomy, or surgical blind loop anastomosis (Billroth II or Roux-Y) [9].

### 2.4. Treatment

To ensure timely management, it is essential to include air embolism in the differential diagnosis of adverse events associated with postoperative ERCP, particularly in patients with known risk factors and acute cardiopulmonary impairment exacerbated in the supine position. If air embolism is suspected, the following steps can have a significant impact on patient outcomes [6].

1. If possible, immediately stop the procedure.
2. Administrate high-flow oxygen at 100%, to reduce the air piston size.
3. Place the patient in lateral decubitus and Trendelenburg position to improve venous return.
4. Perform an emergency echocardiogram.
5. If air is detected on the right side of the heart in the echocardiography, insert a central catheter.
6. Insert a pulmonary artery catheter, if available and if the medical personnel have enough training in its placement and further interpretation.
7. Decompression with nasogastric suction.
8. Start hyperbaric oxygen therapy as soon as the patient's condition allows it—subject to availability.

Once the patient is hemodynamically stable, a brain and thoracic CT scan should be considered to confirm the diagnosis.

### 2.5. Prevention and Prognosis

The use of carbon dioxide (CO_2_) in patients without severe lung disease, instead of air insufflation during ERCP, can eliminate the risk of an air embolism, since the CO_2_ can be easily absorbed [10]. In the aforementioned case, all endoscopies are done with CO_2._

Ideally, the presence of right-to-left shunt should be ruled out in all patients scheduled for ERCP. However, if patients are subject to this procedure there must be immediate availability and expertise to introduce a pulmonary artery catheter in order to drain air acutely from the right atrium. Another suggestion for high-risk patients is to use a precordial Doppler monitor probe during the procedure to quickly detect the presence of air within the heart or pulmonary circulation.

At present, the development of new noninvasive imaging such as endoscopic ultrasound and magnetic resonance cholangiopancreatography (MRCP) has largely replaced ERCP as a diagnostic tool. This will lessen the adverse effects associated with ERCP, which should be restricted to cases with absolute indications [11]. In the present case, prior MRCP was done which justified an ERCP for biliary stent replacement and gallstone removal.

The clinician should keep in mind that the clinical outcome depends largely on the amount and extent of air migration into the veins and that systemic air embolism is associated with cerebral gas embolism, which can have a fatal prognosis.

## 3. Conclusion

Although regional or systemic air embolism is a rare complication associated with ERCP, it should be included in the differential diagnosis in patients with cardiac arrest, cardiogenic shock, respiratory failure, or coma. Treatment strategies include administration of 100% oxygen, nasogastric decompression, placement of the patient in the lateral decubitus and Trendelenburg position, air aspiration via central venous catheter, and hyperbaric oxygen therapy. The outcome in these cases depends on the possibility of establishing an appropriate and early diagnosis in order to start timely measures and reduce mortality.
